# The expression profile of lung long non-coding RNAs and mRNAs in a mouse model of smoke inhalation injury

**DOI:** 10.1080/21655979.2022.2037922

**Published:** 2022-02-13

**Authors:** Zheng-Ying Jiang, Ming-Zhuo Liu, Zhong-Hua Fu, Xin-Cheng Liao, Bin Xu, Liang-Liang Shi, Jia-Qi Li, Guang-Hua Guo

**Affiliations:** Department of Burn, The First Affiliated Hospital of Nanchang University, Nanchang, P. R. China

**Keywords:** Smoke inhalation injury, mouse, long non-coding RNA, competing endogenous RNA

## Abstract

To study the potential expression of lung long non-coding RNAs (lncRNAs) and mRNAs during smoke inhalation injury (SII), using a SII mouse model that we created in our previous work. Microarray was used to investigate the lncRNAs and mRNAs profiles. A bioinformatics analysis was performed. Changes in the top 10 down-regulated and 10 up-regulated lncRNAs were validated using Quantitative Reverse Transcription-PCR (RT-qPCR). The acute lung injury (ALI) mouse model was successfully induced by smoke inhalation, as confirmed by the aberrantly modified cell numbers of red blood cells and neutrophils counts, increased levels of TNF-α, IL-1β, Bax, caspase-7, caspase-3, and decreased Bcl-2 content in lung tissues. When compared to the control mice, 577 lncRNAs and 517 mRNAs were found to be aberrantly expressed in the SII mice. According to the Gene ontology (GO) and Kyoto Encyclopedia of Genes and Genomes (KEGG) pathway analyses, the altered mRNAs were enriched in acute-phase response, oxidoreductase activity, oxidation-reduction process, glutathione metabolism, the wnt signaling pathway, and ferroptosis. A lncRNA-related competitive endogenous RNA (ceRNA) network, including 383 lncRNAs, 318 MicroRNAs (miRNAs), and 421 mRNAs specific to SII, was established. The changes in NONMMUT026843.2, NONMMUT065071.2, ENSMUST00000235858.1, NONMMUT131395.1, NONMMUT122516.1, NONMMUT057916.2, and NONMMUT013388.2 in the lung matched the microarray results. Our findings help to provide a more comprehensive understanding of the pathogenesis of SII as well as new insights into potential therapeutic targets.

## GRAPHICAL ABSTRACT

**Figure:**
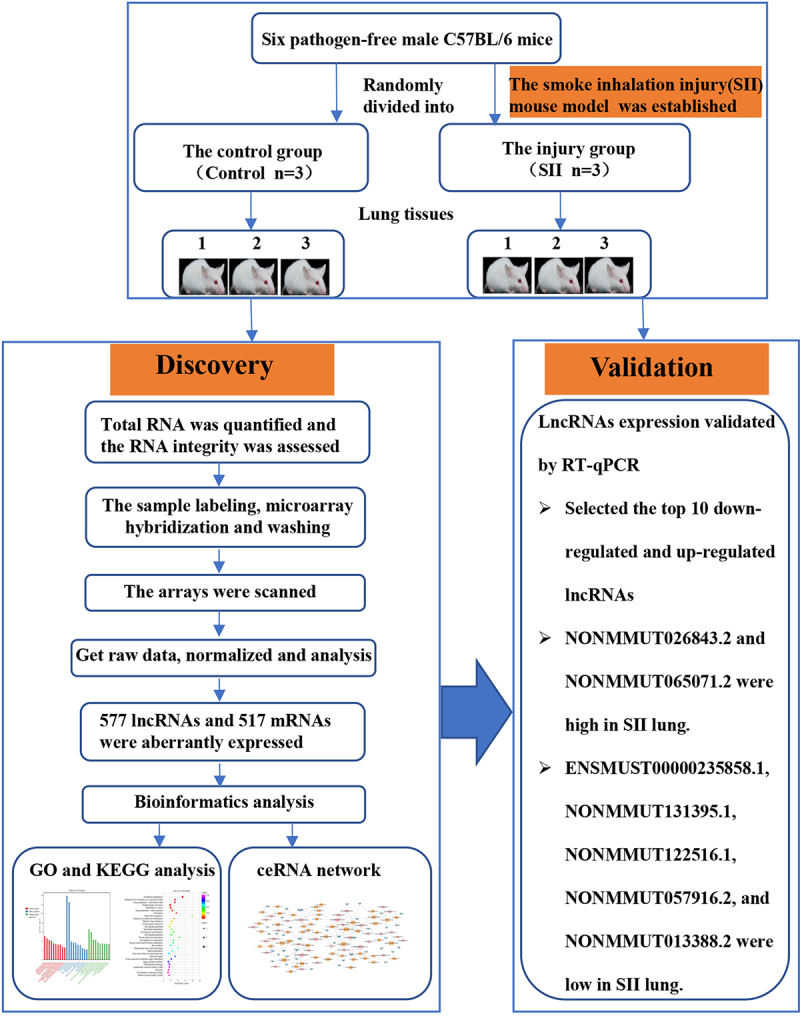

## Introduction

Smoke inhalation injury (SII) is defined as airway and parenchymal injury induced by smoke inhalation involving thermal or chemical irritants. It mainly appears as supraglottic thermal injury, systemic poisoning from absorbed small molecule toxins, chemical irritation of the respiratory tract, or a combination of these insults [1,2]. Patients with SII account for 10.3% of all burn patients; moreover, burns aggravate SII, which increases morbidity and mortality [2]. Furthermore, the incidence of SII is correlated with a central facial burn, an increase in total body surface area of burn, mass casualties, and large-scale fires. Therefore, more than 60% SII occur in patients with central facial burn [3], while 14% of patients with 80–89% of total body surface area burns have SII [4]. The SII-induced inflammatory response may increase fluid resuscitation requirements and the occurrence of pulmonary complications such as pulmonary dysfunction, pneumonia, and acute respiratory distress syndrome (ARDS). Moreover, evidence suggests that the presence of SII in burn patients from fire tragedies increases mortality by nearly 24-fold [5]. Despite remarkable advances in the treatment of burn patients, clinical treatment of SII is still mostly supportive, and targeted therapies remain limited.

Long non-coding RNAs (lncRNAs) are a novel type of non-coding RNAs that are longer than 200 nucleotides and can regulate gene expression at different levels, such as epigenetic, transcriptional, and post-transcriptional [6]. Furthermore, many studies have indicated that lncRNAs play key functions in numerous diseases, including cardiac and infectious diseases, rheumatic diseases, and cancers [7–11]. Salmena et al. [12] reported for the first time that lncRNA acts as ceRNA and that lncRNA, mRNA, and other types of RNAs acting as natural miRNA sponges can regulate the function of target miRNAs via shared microRNA response elements. Furthermore, a growing body of evidence indicates that lncRNA acting as ceRNA are involved in the progression of many disorders [13–15]. Taken together, the role of lncRNAs in the progression of many diseases is being increasingly recognized. Nevertheless, to the best of our knowledge, no previous research on the comprehensive landscape of lncRNA-related ceRNA regulatory networks in SII has been conducted. In this study, we established a mouse model of SII based on our previous work [16,17], which was evidenced by changes in apoptosis, inflammatory factors, and pathophysiology in lung tissues. We aimed to profile and quantify differentially expressed lncRNAs and mRNAs in the lung between the SII mice and normal control mice using Microarray technology, and provide new insights into the fundamental pathological processes underlying SII using bioinformatics approaches. We anticipate that our findings will be informative for future studies into the progression and therapeutic targets of SII.

## Materials and methods

### Animals

Six pathogen-free male C57BL/6 mice (8–10 weeks old, 23–27 g) were purchased from the experimental animal center of Nanchang University. The mice were randomly divided into two groups: the control group and the injury group (n = 3 in each group). Mice were caged in a room with a temperature of 22 ± 1°C and 50% relative humidity. All experimental procedures in this study were approved by the Laboratory Animal Ethics Committee of First Affiliated Hospital of Nanchang University.

### Smoke inhalation injury model preparation

The SII mouse model was created using a self-made smoke generator [16,17]. First, conscious mice from the injury group were isolated in a mouse cage. The cage was then placed in a smoke box of the self-made smoke generator, and smoldering wood sawdust was used to produce smoke (150 g/kg, sawdust/body weight), which was released into the smoke box via a manual valve. The mice were exposed to the smoke for 45 seconds before being exposed to ambient air for 60 seconds. The procedure was repeated four times. After 24 h, the mice were executed by injecting an overdose of sodium pentobarbital (150 mg/kg body weight) intraperitoneally, and lung tissues were collected. The control group received the same treatment as the injury group, except for smoke inhalation.

### Histopathological evaluation

The upper lobe of the right lung tissues were infiltrated in 4% formalin, dried, and finally embedded in paraffin. The lung tissues were then cut into 5 mm thick sections and stained with hematoxylin and eosin (HE) staining [16]. Furthermore, two pathologists conducted a double-blind analysis on the lung histopathological changes.

### Western blot analysis

Total protein was extracted and separated using sodium dodecyl sulfate polyacrylamide gel electrophoresis (SDS-PAGE) from the middle lobe of the right lung tissues. After being electrobotted onto polyvinylidene fluoride (PVDF) membranes, the membranes were blocked with 5% skim milk and placed at room temperature for one hour before being hybridized with the following primary antibodies: IL-1β, caspase-3, Bcl-2, and Bax (Abcam, USA); and TNF-α, caspase-7, and GAPDH (Proteintech, China). After an overnight incubation at 4°C, the membranes were washed three times with 1 × TBST buffer. After incubation with the HRP-labeled secondary antibody for one hour, the membranes were washed three times for 10 minutes each with 1 × TBST buffer. Finally, the membranes were treated with enhanced chemiluminescence (ECL) reagent and exposed in the darkroom to evaluate the expression levels of the target protein [16].

### Transcriptomic microarray and computational analysis

We used an Agilent Mouse ceRNA Microarray 2019 (4 * 180 K, Design ID: 086242) to investigate changes in lncRNAs and mRNAs expression through OE Biotech Co., Ltd. (Shanghai, China). The microarray data was also subjected to bioinformatics analysis. The raw data was normalized using a quantile algorithm. Then, we identified differentially expressed lncRNAs and mRNAs between the injury and the control groups using absolute fold change and P value. An absolute fold change ≥ 2.0 and P value ≤ 0.05 were used as the thresholds of up- and down-regulated genes. The underlying functions of the differentially expressed mRNAs were then investigated using Gene Ontology (GO) and Kyoto Encyclopedia of Genes and Genomes (KEGG) analyses [18]. The ceRNA network was constructed to better understand the underlying functions of the differentially expressed lncRNAs and mRNAs in SII mice, with the hypothesis that lncRNAs can act as natural miRNA sponges, directly modulating the expressional level of miRNAs and mRNA and being involved in posttranscriptional regulation. The differentially expressed lncRNA, which was significantly positively correlated with the differentially expressed mRNA, was selected as the lncRNA-targe for ceRNA analysis. All known miRNAs of the species in the database mirbase22 were used to predict the microRNA-targe, which significantly correlated with the lncRNA-targe and the differentially expressed mRNA. Target binding sites were used to predict lncRNA-miRNA and miRNA-mRNA pairs, which were also filtered by coexpression correlation of microRNA-targe. The ceRNA network was created with lncRNA-mRNA pairs that share miRNA and have the same regulating direction [19].

### Validation of differentially expressed lncRNAs by quantitative reverse transcription-PCR (RT-qPCR)

Based on microarray results, the top 10 downregulated and upregulated lncRNAs were verified using RT-qPCR. The internal reference was GAPDH, and the target primers were designed using Primer software (Table 1). Relative gene expression levels were calculated using the 2^−∆∆Ct^ method [20].

**Table 1.** The primers used in this study

Name	Primers (5’- 3’)	Product length (bp)
NONMMUT143086.1	F:GCTTAGGACCTCACTGAAAATC	108
	R:GGCAGGGAGTAAGAGAGACA	
NONMMUT093631.1	F:ATGTAGTTGTGGGTGGCT	83
	R:ACCAAGACAGTGTTCCCTC	
NONMMUT032726.2	F:TCTCTGCCTGGAATAGGGCA	86
	R:TTCCTTTCTTCTCATGATAGGT	
NONMMUT057421.2	F:CCAGTTCCCATGTTACACAAT	112
	R:TGCATGAGACTGTGACTCC	
NONMMUT026843.2	F:GGACAGAGTGGTAAAGAGAGAA	113
	R:GACAGTTGTCCCTGGGAA	
NONMMUT001456.2	F:CCACCATCACCCCATATACT	97
	R:GTTCCTATATGGCCCATCCGA	
NONMMUT065071.2	F:ACATATCTTCTGTGGCTTAACC	80
	R:TGCATCCTAGTCTGTATGCG	
ENSMUST00000223214.1	F:CCAGAAACTGCGACAATGACA	83
	R:ATCTACCAATCTATGCAAAA	
ENSMUST00000221685.1	F:CCCACAGTGACCTAGCAT	88
	R:CAAAGCCTTACCCACGTATT	
NONMMUT057416.2	F:GACATTTCCACCAGGACTC	85
	R:AGGCCCATTCATTCTTACC	
ENSMUST00000195997.1	F:ACAGAGACACTACACGGT	93
	R:TCAGTCTCCATTCTGCTGAT	
NONMMUT095883.1	F:TCTCATCAGAAGGCCCTTGAA	87
	R:CTCAGTGCTTGGCACATAG	
NONMMUT131395.1	F:AGAATGAGGAGGCTGGAC	82
	R:CTCACACTAGAAGGGCTAGAT	
NONMMUT018978.2	F:AAACTGTCAACTTGGTACTCAT	110
	R:CACCTACTCCAACAAAGCC	
ENSMUST00000061899.5	F:CTCAGATGCTGTGCTTGG	90
	R:AATCAATACGCAGGCACC	
NONMMUT013388.2	F:ACTGCGTGGAGTGGATTA	124
	R:CTTGTGTCTGTGTCCAAATG	
NONMMUT062594.2	F:CTCCCTCATATTTTGTTTTATA	88
	R:GCAGAGTGTTTTCTGCTG	
ENSMUST00000235858.1	F:AGAGATCCTGTTCTCAAGCA	80
	R:GAAGAGAAACATCAGGTGTCC	
NONMMUT122516.1	F:ACAACAGCATCATTGGCTC	98
	R:AGATGGCCCTCTGATTACC	
NONMMUT057916.2	F:TTCTGCTTAGAAACTCACCAAC	87
	R:TGAAGTCAGGTCCTTGACTAC	
GAPDH	F:GCAAGGACACTGAGCAAGA	76
	R:GGATGGAAATTGTGAGGGAG	

### Statistical analysis

All statistical analyses were performed using SPSS version 17.0. All data are expressed as the mean ± SD. The differences between control and SII mice were analyzed using a one-way ANOVA or a student’s t test. Statistical significance was considered when P < 0.05.

## Results

### Establishment of SII mouse model

**Figure 1.**
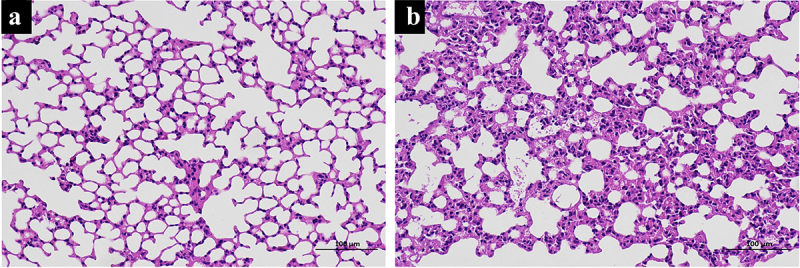
Histologic changes in mouse lung tissue. The histologic changes in control mice (a) and SII mice (b) were examined using HE-staining.

**Figure 2.**
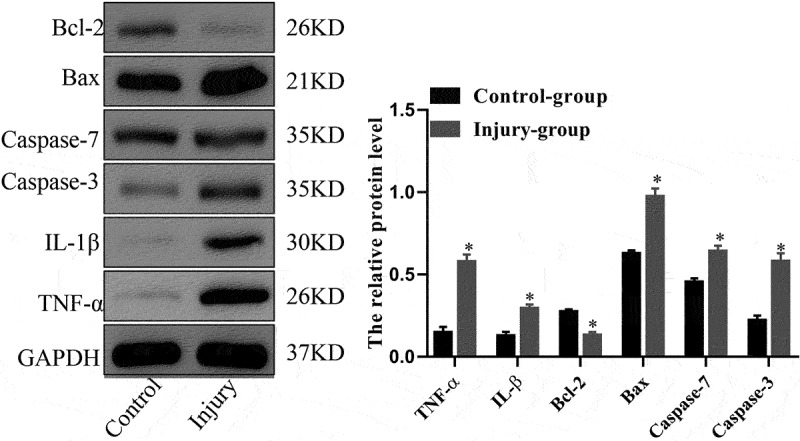
The protein expression levels of TNF-α, IL-1β, Bcl-2, Bax, caspase-7 and caspase-3 in mouse lung as assessed using Western blot analysis. *P < 0.05 compared with control.

The SII model was created using a self-made smoke generator based on previous work. There was evident apoptosis and inflammation in the lungs of the SII mice at 24 h after smoke inhalation. A large amount of inflammatory cell infiltration, diffuse hemorrhage, dramatic edema, and partial alveolar septum thickening in lungs of SII mice was revealed by HE-staining (Figures 1(a,b)). In the SII group, the levels of IL-1β, TNF-α, Bax, caspase-7, and caspase-3 in the lung increased significantly, while the levels of Bcl-2 decreased markedly (P < 0.05; Figure 2). These findings indicated that the SII mouse model had been successfully created.

### LncRNA and mRNA expression profile

**Figure 3.**
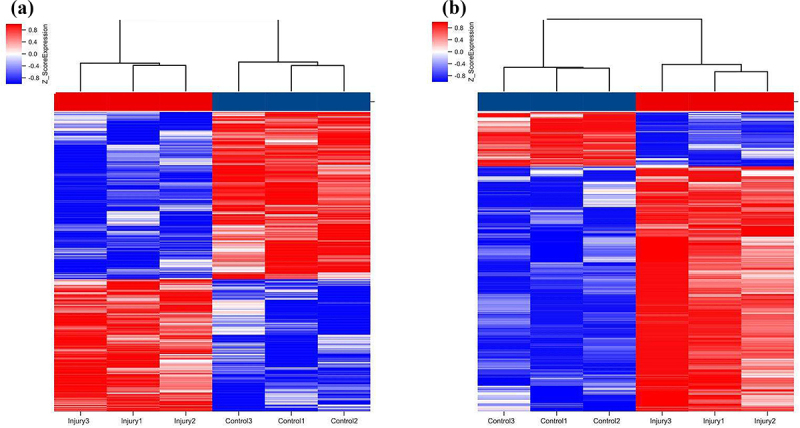
Hierarchical clustering of lncRNAs (a) and mRNAs (b) differentially expressed in the lung of smoke inhalation injury mice versus control mice. Heat maps showing significantly (absolute fold-change ≥ 2.0, P ≤ 0.05) regulated lncRNAs (a) and mRNAs (b). Three mice were analyzed in each group. Expression values are presented with the intensity of the color scheme, which ranges from blue to red, indicating low to high relative expression, respectively.

We used the Agilent Mouse ceRNA Microarray to identify lung lncRNAs and mRNAs in the lungs of SII and normal control mice to explore the potential mechanisms involved in SII pathogenesis. We identified 577 differentially expressed lncRNAs in the lungs of mice between the SII and the control groups, with 322 significantly downregulated and 255 significantly upregulated (absolute fold-change ≥ 2.0 and P ≤ 0.05), as shown in Figure 3(a). The most downregulated and upregulated lncRNAs were ENSMUST00000195997.1 (absolute fold-change = 18.30, P = 0.0006) and NONMMUT143086.1 (absolute fold-change = 9.52, P = 0.0099), respectively. The top 10 downregulated and 10 upregulated lncRNAs identified via the microarray analysis are shown in Table 2.

**Table 2.** The detailed information of the top ten up-regulated and top ten down-regulated lncRNAs

No.	Probe ID	Seqname	Chromosome	RNA length	Regulation	Fold change	P-value
1	mclstr_9021_4318_15	NONMMUT143086.1	chr14	4622	up	9.521910894	0.009859008
2	mclstr_117730_422_11	NONMMUT093631.1	chr14	663	up	9.388565601	0.006732134
3	mclstr_64675_608_22	NONMMUT032726.2	chr18	1442	up	8.863760296	0.007069417
4	mclstr_31310_2100_16	NONMMUT057421.2	chr6	2646	up	7.975907107	0.001080942
5	mclstr_157442_157_12	NONMMUT026843.2	chr16	239	up	7.640112801	0.00339818
6	mclstr_25509_473_8	NONMMUT001456.2	chr1	2958	up	7.446228814	0.00126251
7	mclstr_132992_391_18	NONMMUT065071.2	chr8	508	up	7.212371845	0.009701478
8	mclstr_155799_175_1005	ENSMUST00000223214.1	chr12	259	up	6.467383032	0.018115792
9	mclstr_124549_507_10	ENSMUST00000221685.1	chr12	604	up	6.32425502	0.006779035
10	mclstr_96138_617_16	NONMMUT057416.2	chr6	884	up	6.163481235	0.000390771
11	mclstr_38339_342_10	ENSMUST00000195997.1	chr3	2312	down	−18.30478859	0.000577554
12	mclstr_114790_507_17	NONMMUT095883.1	chr15	689	down	−10.45263266	0.00053615
13	mclstr_16724_1241_8	NONMMUT131395.1	chr8	3608	down	−9.425183297	0.001148963
14	mclstr_57822_1101_16	NONMMUT018978.2	chr13	1621	down	−9.087390444	0.001534071
15	mclstr_45697_949_6	ENSMUST00000061899.5	chr13	2012	down	−8.597166648	0.000508943
16	mclstr_13889_2858_12	NONMMUT013388.2	chr12	3902	down	−8.293207867	0.000776749
17	mclstr_150929_111_18	NONMMUT062594.2	chr7	320	down	−7.770474462	0.000520666
18	mclstr_61252_1265_10	ENSMUST00000235858.1	chr17	1528	down	−6.734403486	0.001872791
19	mclstr_114748_240_18	NONMMUT122516.1	chr6	689	down	−6.275645719	0.000285554
20	mclstr_61965_1408_5	NONMMUT057916.2	chr6	1510	down	−6.015964281	0.00112245

There are top ten up-regulated and top ten down-regulated lncRNAs between smoke inhalation injury mice and controls.

In the lungs of SII mice, 106 mRNAs were downregulated and 411 mRNAs were upregulated (absolute fold-change ≥ 2.0 and P ≤ 0.05) compared to the control group (Figure 3(b)). The most downregulated and upregulated mRNAs were Dlk1 (absolute fold change = 10.14, P = 0.0009) and Sprr1a (absolute fold change = 107.13, P = 0.0014), respectively. The top 10 downregulated and 10 upregulated mRNAs identified via the microarray analysis are shown in Table 3.

**Table 3.** The detailed information of the top ten up-regulated and top ten down-regulated mRNAs

No.	Probe ID	Seqname	Chromosome	RNA length	Regulation	Fold change	P-value
1	mclstr_102710_549_14	ENSMUST00000054599.7	chr3	806	up	107.1279464	0.001410693
2	mclstr_96820_749_19	ENSMUST00000098537.3	chr9	875	up	60.14868173	1.68E-05
3	mclstr_114048_331_8	ENSMUST00000122979.1	chr5	695	up	49.03799846	0.000401458
4	mclstr_55441_1109_10	ENSMUST00000081277.8	chr1	1689	up	36.38834831	0.00083363
5	mclstr_80211_1012_18	ENSMUST00000111137.7	chr5	1119	up	35.25404877	9.76E-05
6	mclstr_36002_1370_4	ENSMUST00000194462.5	chr3	2419	up	31.38127593	0.000423606
7	mclstr_63893_697_6	ENSMUST00000105683.8	chr4	1460	up	30.33530503	0.001345788
8	mclstr_102453_400_26	ENSMUST00000125479.7	chr9	809	up	28.58030453	6.74E-05
9	mclstr_30065_738_5	ENSMUST00000033806.4	chrX	2707	up	25.01589362	6.99E-05
10	mclstr_113967_323_10	ENSMUST00000023328.7	chr16	696	up	24.2123413	0.000939283
11	mclstr_13777_3038_12	ENSMUST00000173539.7	chr12	3914	down	−10.13917004	0.000929047
12	mclstr_102799_397_14	ENSMUST00000177159.8	chr6	805	down	−9.633130364	0.001313476
13	mclstr_61833_244_7	ENSMUST00000002663.11	chr6	1513	down	−8.802692292	0.000811013
14	mclstr_44782_1545_7	ENSMUST00000028010.8	chr1	2046	down	−8.780208895	0.009062684
15	mclstr_122776_156_17	ENSMUST00000176945.1	chr6	619	down	−8.701782504	0.000258663
16	mclstr_18474_2678_15	ENSMUST00000190247.6	chr12	3458	down	−7.633113125	0.000989546
17	mclstr_57261_847_6	ENSMUST00000238049.1	chr19	1637	down	−7.3432932	0.003232176
18	mclstr_140398_25_1007	ENSMUST00000237732.1	chr17	429	down	−6.691039967	0.001705893
19	mclstr_60995_1145_8	ENSMUST00000236584.1	chr17	1535	down	−6.681386222	0.001423149
20	mclstr_55806_1449_5	ENSMUST00000101513.8	chr5	1679	down	−6.21342964	0.017611573

There are top ten up-regulated and top ten down-regulated mRNAs between smoke inhalation injury mice and controls.

### GO and KEGG analysis of differentially expressed genes

**Figure 4.**
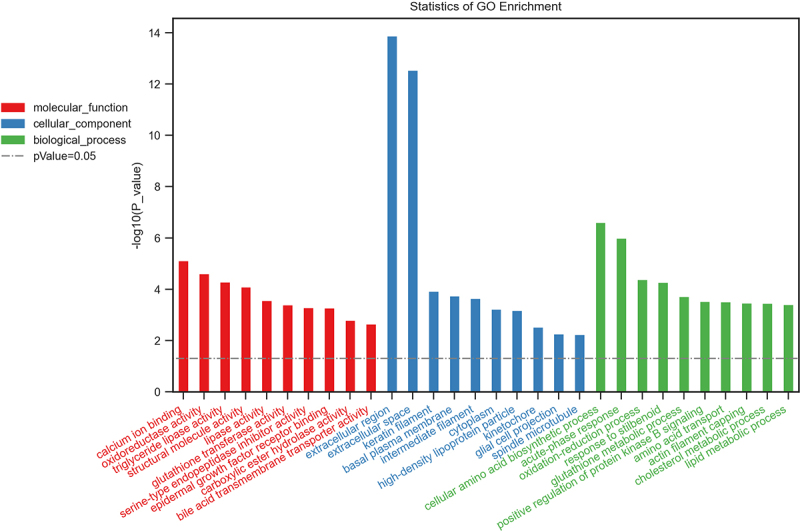
Biological functions of differentially expressed mRNAs. The top 10 enriched GO categories involved in molecular function, cellular component, and biological processes.

To gain further insights into the functions of differentially expressed genes, GO enrichment analysis and KEGG pathway analysis were conducted. According to GO analysis, the top three enriched molecular functions that could regulate genes were calcium ion binding (Term ID: 0005509), oxidoreductase activity (Term ID: 0016491), and triglyceride lipase activity (Term ID: 0004806); the top three enriched cellular components that could regulate genes were extracellular region (Term ID: 0005576), extracellular space (Term ID: 0005615), and keratin filament (Term ID:0045095); and the top three enriched biological processes that could regulate genes were cellular amino acid biosynthetic process (Term ID: 0008652), acute-phase response (Term ID: 0006953), and oxidation-reduction process (Term ID: 0055114) (Figure 4).

**Figure 5.**
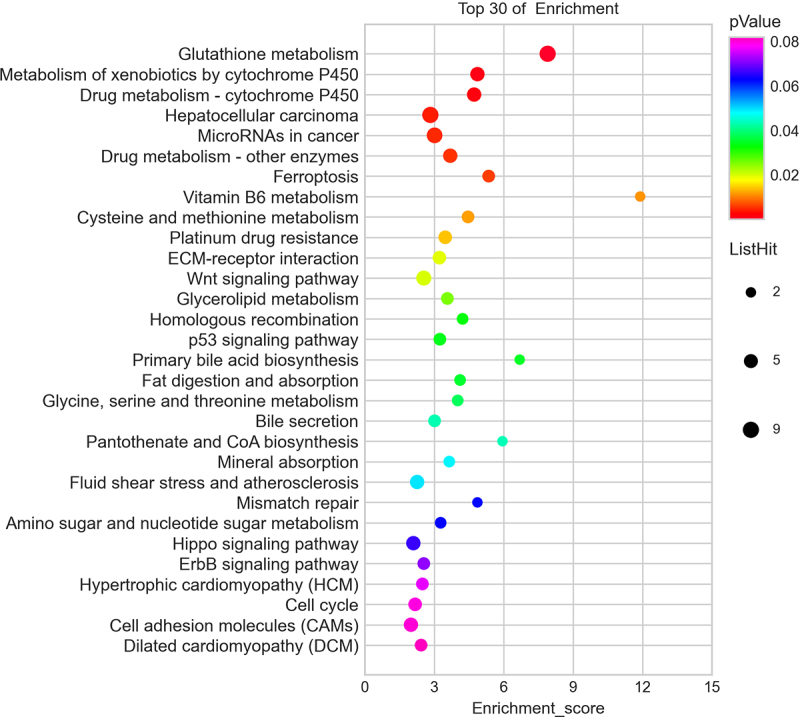
Pathway analysis for differentially expressed mRNAs. The top 30 most enriched pathways based on the KEGG pathway analysis.

According to the KEGG analysis, the main signal pathways that could regulate genes were glutathione metabolism, ferroptosis, drug metabolism -cytochrome P450, metabolism of xenobiotics by cytochrome P450, hepatocellular carcinoma, microRNAs in cancer, and drug metabolism -other enzymes (Figure 5).

### LncRNA-miRNA-mRNA ceRNA network analysis

**Figure 6.**
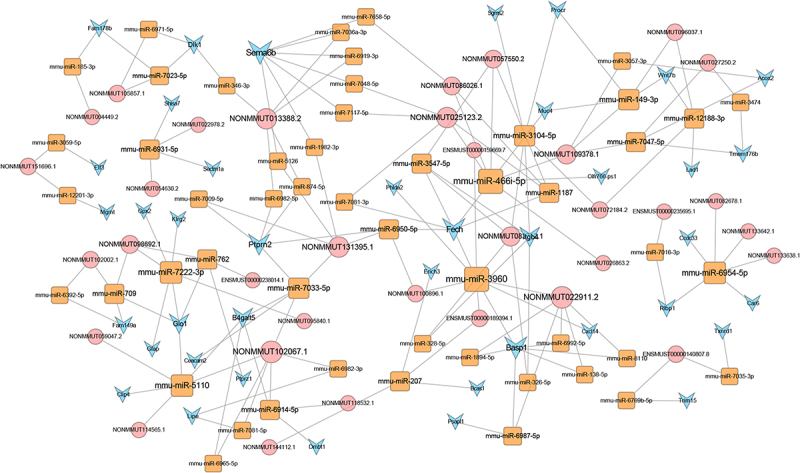
LncRNA-miRNA-mRNA ceRNA network in smoke inhalation injury mouse. The diamond, rectangle, and triangle represent differentially expressed lncRNAs, miRNAs, and mRNAs, respectively.

To better understand the effects of lncRNAs on mRNAs mediated by combination with miRNAs in SII, we built a ceRNA network based on the aforementioned data. Among the proposed ceRNA network, 5122 lncRNA-miRNA-mRNA interaction pairs including 383 lncRNAs, 318 miRNAs, and 421 mRNAs were identified. The ceRNA regulatory network of lncRNA-miRNA-mRNA, including the top 100 interaction pairs, was established using Cytoscape (version 3.7.0) (Figure 6).

### LncRNAs expression validated using RT-qPCR

**Figure 7.**
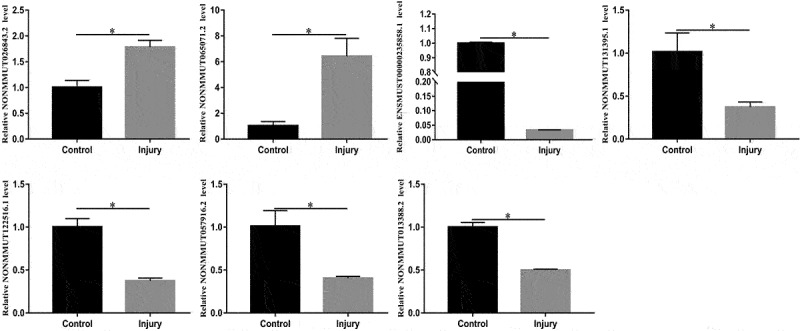
Relative expression levels of NONMMUT026843.2, NONMMUT065071.2, ENSMUST00000235858.1, NONMMUT131395.1, NONMMUT122516.1, NONMMUT057916.2, and NONMMUT013388.2 in the lung. The candidate LncRNAs were verified using RT-qPCR. *P < 0.05 versus control.

To validate the reliability of Microarray data, RT-qPCR were conducted. The top 10 downregulated and 10 upregulated lncRNAs were selected and subsequently validated using RT-qPCR. The results of the RT-qPCR validation assay indicated that changes in seven lncRNAs were congruent to the results of the Microarray (Figure 7).

## Discussion

To develop new therapeutic strategies, a thorough understanding of the pathophysiology and potential molecular mechanisms of acute lung injury (ALI) induced by smoke inhalation is imperative. In this study, we developed a self-made smoke generator and created a SII mouse model based on our previous research [16,17]. Subsequently, the SII mouse model was used to identify lncRNAs and investigate their roles.

In recent years, the function of lncRNAs in the research of lung diseases has received increased attention. Many studies have indicated that lncRNAs are involved in a variety of lung diseases, including pulmonary fibrosis, lung cancer, ALI, and so on [14,21,22]. Wang et al. found that plasma MALAT1 expression was significantly high in ARDS patients and that it induced cell apoptosis during ARDS pathogenesis [23]. However, their research only involved a single genetic event, and might not have met the current requirement for identifying underlying molecular therapeutic targets for SII. Many studies on sequencing or transcriptome microarray have demonstrated that numerous differentially expressed lncRNAs play a unique role in the progression of multiple diseases [7–11]. Wang et al. discovered a possible link between lncRNAs changes and lipopolysaccharide (LPS)-induced ALI using lncRNAs microarray analysis. In their study, 2632 lncRNAs, including 1418 downregulated lncRNAs and 1214 upregulated lncRNAs, were differentially expressed in mice between the LPS-induced ALI group and the control group. Moreover, uc007pnu.1, ENSMUST00000016031.13, and ENSMUST00000170214.1 were found to be upregulated, and found the interfering of ENSMUST00000016031.13 and ENSMUST000001-70214.1 markedly decreased the contents of IL-1β and TNF-α induced by LPS [20].

In this study, we used Microarray to evaluate the lung tissue lncRNAs and mRNAs profiles in mice with SII and identified 322 downregulated lncRNAs and 255 upregulated lncRNAs, 106 downregulated mRNAs and 411 upregulated mRNAs in the lungs of mice with SII compared to the control group. Our microarray results of the top 10 downregulated and 10 upregulated lncRNAs were validated using RT-qPCR. The RT-qPCR data showed that the changes in NONMMUT026843.2, NONMMUT065071.2, ENSMUST00000235858.1, NONMMUT131395.1, NONMMUT122516.1, NONMMUT057916.2, and NONMMUT013388.2 were consistent with those found in the microarray assay. Subsequently, we conducted further research on the identified differentially expressed lncRNAs and potential molecular mechanisms.

Many studies have demonstrated that lncRNAs are involved in numerous physiologic or pathologic processes, such as chromatin remodeling, protein expression, epigenetic regulation, and so on [24]. Accordingly, GO analyses were performed to investigate the underlying functions of the differentially expressed mRNAs. The results showed that the differentially expressed mRNAs were mainly involved in acute-phase response, oxidoreductase activity, and oxidation-reduction process. Acute-phase response is an innate systemic response to tissue damage, infection, and environmental insults such as smoke, and it refers to the rapid host reaction induced by several overlapping inflammatory pathways. Several proinflammatory cytokines, including TNF-α, IL-6, and IL-1, have been associated with the initiation of the acute phase response [25,26]. Furthermore, the synthesis and secretion of acute phase proteins induced by the mentioned mediators may either inhibit or enhance inflammatory processes. Previous research has revealed that C-reactive protein is the most useful indicator of an acute-phase response among all acute-phase proteins; additionally, the treatment of an acute-phase response is to address its etiology [27]. Reactive oxygen species (ROS) are significantly increased after inhalation injury. Common ROS include hydroxyl radicals (OH−), superoxide anions, and hydrogen peroxide (H2O2) [28]. These factors, which include bronchoconstriction, nitric oxide synthase (NOS), and histamine, contribute to an increase in ROS production [29,30]. Reactive oxygen species appear to play important roles in inhalation injury models, including causing the dysfunction of mitochondria and cellular apoptosis, fluid loss, and plasma proteins extravasating from the intravascular space into alveoli and bronchioles, as well as cell injury via cytokine production and neutrophil attraction [31,32]. Furthermore, one of the characteristics of inflammatory lung diseases is an oxidant/antioxidant imbalance, which can lead to cell damage [33]. Indeed, Park et al. demonstrated that changes in other indices of oxidative stress were more variable and significant in plasma and lung tissue after SII [34].

According to the KEGG analysis, the identified differentially expressed genes were mainly involved in glutathione metabolism, ferroptosis, and the wnt signaling pathway. Glutathione can be defined as a critical protective antioxidant that has been proven to participate in inflammatory responses and immune modulation [33]. Moreover, glutathione has been found to protect airspace epithelium from cigarette smoke (free radicals/air particulates)-mediated inflammation and damage [35,36]. Changes in GSH metabolism in alveolar and lung are extensively considered to be a key feature of a variety of lung diseases, such as ARDS [37], NO_2_-induced acute and chronic lung injury [38], chronic obstructive pulmonary disease [39], and asthma [40].

Ferroptosis, the most recently discovered mode of nonapoptotic cell death, has an important role in the progression of LPS-induced ALI [41], and intestinal ischemia/reperfusion-induced ALI [42]. A recent study reported that lncRNA LINC00336 inhibits ferroptosis in lung cancer by regulating the expression of cystathionine-β-synthase [43]. Previous studies have indicated that the wnt signaling pathway has a critical function in LPS-induced ALI models [44,45].

Recent research has revealed that the lncRNA and ceRNA network plays a critical function in the progression of various disorders [46,47]. According to Dai et al., knocking down the LncRNA MALAT1 inhibited the inflammatory response in LPS-induced ALI by functioning as a ceRNA [48]. Qiu et al. found that LncRNA TUG1 acted as a ceRNA to alleviate sepsis-induced inflammation and apoptosis in sepsis-induced ALI [49]. Our study identified 5122 lncRNA-miRNA-mRNA interaction pairs in the proposed ceRNA network, including 383 lncRNAs, 318 miRNAs, and 421 mRNAs. Notably, 27 upregulated lncRNAs were positively associated with tribbles homolog 3 (TRIB3). In lung cancer cells, TRIB3 has been found to regulate cell apoptosis by activating nuclear factor-κB signaling [50]. The current study implied that lncRNAs might promote cell apoptosis and inflammatory responses in SII by functioning as ceRNA. Further studies are needed to investigate the potential roles of the differentially expressed lncRNAs in the progression of SII.

## Conclusions

In conclusion, hundreds of lncRNAs and mRNAs were found to be differentially expressed in the lungs of the SII mice based on the model. Bioinformatics analyses showed that differentially expressed lncRNAs may have a variety of underlying functions related to differentially expressed genes. Moreover, acute-phase response, oxidoreductase activity, oxidation-reduction process, glutathione metabolism, ferroptosis, and the wnt signaling pathway may play key roles in the progression of SII. The use of lncRNA as ceRNA may contribute in clarifying the underlying roles of lncRNAs in the molecular mechanisms and treatment of SII, and the seven validated lncRNAs may be used to identify underlying therapeutic targets and diagnose SII. However, additional *in vitro and in vivo* experiments are required to validate our findings.

## Data Availability

The data included in this study can be available from the corresponding author on reasonable request.
